# Supplementary material for: Canonical and non-canonical PRC1 differentially contribute to regulation of neural stem cell fate
Source: Life Sci Alliance. 2025 Feb 11;8(4):e202403006. doi: 10.26508/lsa.202403006 (PMC11814486; doi:10.26508/lsa.202403006)

A uncropped gels related to Fig2 B, C

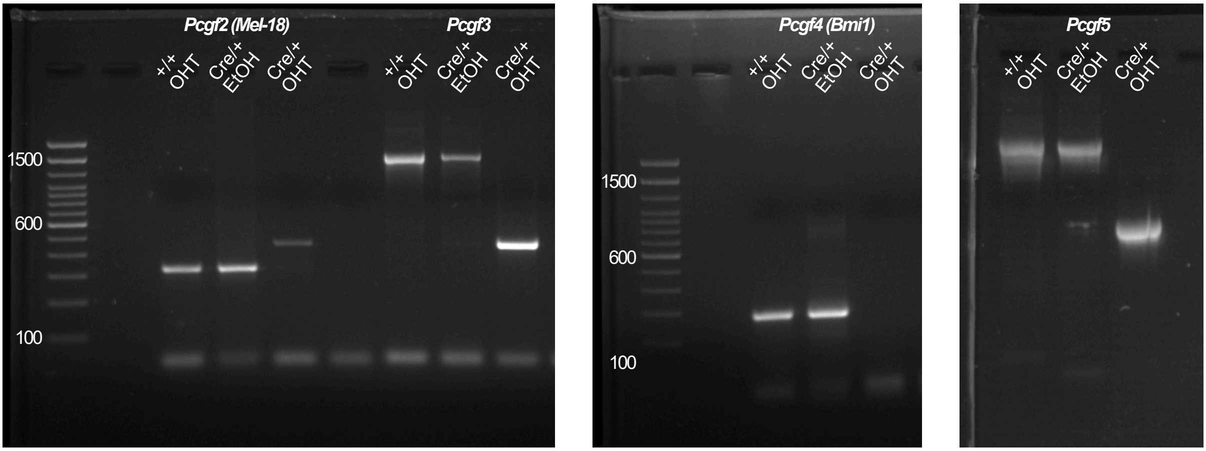

B uncropped blots related to Fig2 D, E

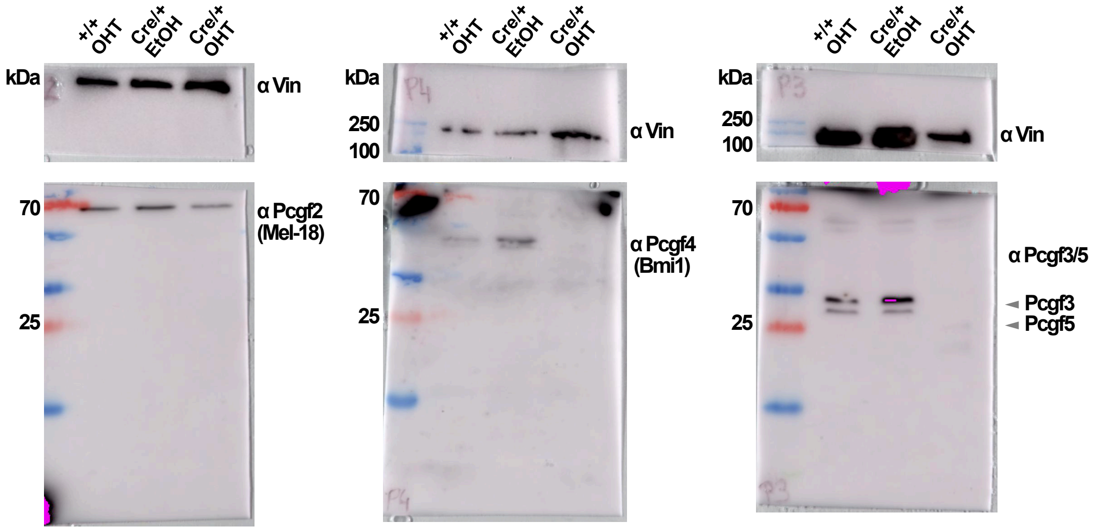

C uncropped blot related to Fig2 F, H

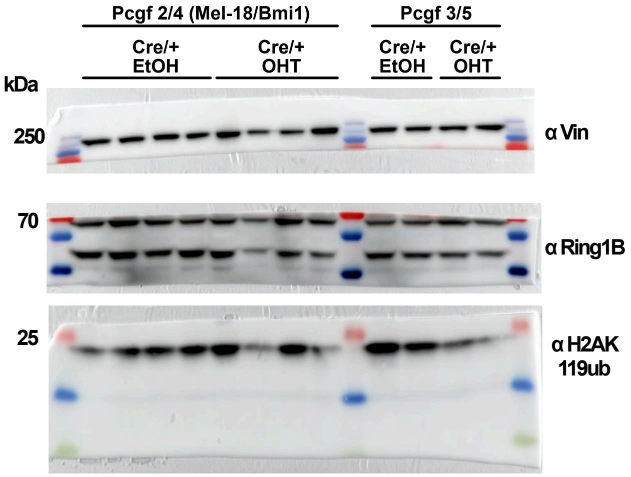

Supplement: Supplementary file 1 [file LSA-2024-03006_SdataF2.pdf]
